# Ultrasonic Monitoring of the Interaction between Cement Matrix and Alkaline Silicate Solution in Self-Healing Systems

**DOI:** 10.3390/ma10010046

**Published:** 2017-01-07

**Authors:** Mohand Ait Ouarabi, Paola Antonaci, Fouad Boubenider, Antonio S. Gliozzi, Marco Scalerandi

**Affiliations:** 1 Department of Applied Science and Technology, Condensed Matter and Complex Systems Physics Institute, Politecnico di Torino, 10129 Torino, Italy; aitouarabi@gmail.com (M.A.O.); antonio.gliozzi@polito.it (A.S.G.); marco.scalerandi@infm.polito.it (M.S.); 2 Laboratoire de Physique des Matériaux, Université des Sciences et de la Technologie Houari Boumediene, BP 32 El Alia, Bab Ezzouar 16111, Algeria; fboubenider@yahoo.fr; 3 Department of Structural, Geotechnical and Building Engineering, Politecnico di Torino, 10129 Torino, Italy

**Keywords:** self-healing concrete, sodium silicate, cracks, NDT, nonlinear ultrasonic inspection, scaling subtraction method

## Abstract

Alkaline solutions, such as sodium, potassium or lithium silicates, appear to be very promising as healing agents for the development of encapsulated self-healing concretes. However, the evolution of their mechanical and acoustic properties in time has not yet been completely clarified, especially regarding their behavior and related kinetics when they are used in the form of a thin layer in contact with a hardened cement matrix. This study aims to monitor, using linear and nonlinear ultrasonic methods, the evolution of a sodium silicate solution interacting with a cement matrix in the presence of localized cracks. The ultrasonic inspection via linear methods revealed that an almost complete recovery of the elastic and acoustic properties occurred within a few days of healing. The nonlinear ultrasonic measurements contributed to provide further insight into the kinetics of the recovery due to the presence of the healing agent. A good regain of mechanical performance was ascertained through flexural tests at the end of the healing process, confirming the suitability of sodium silicate as a healing agent for self-healing cementitious systems.

## 1. Introduction

Due to their attractive potential and practical value, self-healing materials have been extensively investigated over the last ten years, and significant advances have been achieved [1]. In particular, self-healing in cementitious materials has been targeted through different strategies, from enhancing intrinsic healing [2,3] to exploiting bacterial metabolic reactions [4], up to developing novel autonomic vascular [5] or capsule-based systems [6,7,8,9].

Among all of them, the capsule-based approach appears to be one of the most promising due to its versatility. It consists of adding to the concrete mix a variable amount of micro- or macro-capsules that sequester one or more healing agents inside them, in such a way that when the capsules are ruptured by damage, the self-healing mechanism is triggered through the release and reaction of the healing agent(s) in the region of damage. The type of healing agent to be stored in the capsules can be changed according to the desired healing mechanism to be generated, ranging from mono-component systems (in which a single healing agent is present and the healing process is expected to take place upon its reaction with air, or with ambient humidity, or with the cement matrix itself) to multi-component systems (in which two or more healing agents are present, reacting upon contact with each other).

This paper takes its starting point from the observation of mono-component systems that display a lower level of complexity with respect to the multi-component counterpart, also minimizing the risks of incomplete reaction due to insufficient mixing of the healing agents. In particular, attention is focused on mono-component encapsulated systems using an alkaline silicate solution as a healing agent. Indeed, such alkaline solutions as sodium, potassium or lithium silicates are considered to be very promising for the purpose of developing successful self-healing cementitious systems because of their low viscosity, which enables them to effectively diffuse through the cracks once released at the damage site, and also owing to their chemical affinity with the cement matrix. Their good compatibility with cementitious composites is well known by the construction industry, where soluble silicates, in particular sodium silicate (also referred to as “water-glass”), already have several applications. For example, they are used as moisture reducers in the wet kiln process of clinker production [10], as binders for cold consolidation of silica-based aggregates [11], as active ingredients in the production of dense or lightened geopolymers [12] and as soil stabilizers [13,14]. Furthermore, one of their foremost uses is as concrete sealers. Unlike other sealants, soluble sodium silicate penetrates the concrete surface to form new solid species. As a result, the surface has enhanced properties, such as decreased permeability, increased hardness and overall increased durability [10].

Despite their common use in the cement industry, the exact mechanisms by which the silicates act to improve the performance of concretes is still controversial. Excluding the gelation/polymerization reactions that occur rapidly when the pH of liquid silicate falls below 10.7 (which is not the case of the concrete environment, which is known to be highly alkaline), one argument is that sodium silicates behave as efficient sealers because SiO${}_{2}$ precipitates in the pores upon evaporation or absorption of water, thus contributing to densify the concrete microstructure [15]. A second standpoint is that the silicates are able to form an expansive gel similar to that generated during alkali-silicate reactions to fill the concrete voids by swelling [10,16]. A third theory is that the active silicates can react with the excess portlandite or calcium hydroxide (CH) available in the concrete to yield relatively insoluble calcium silicate hydrates (C-S-H gels) [10,16,17,18]. Similar reactions are theorized for potassium and lithium silicates, as well. Various authors agree that the latter is the main mechanism of self-healing by alkaline silicate solutions [8,19,20], because the delayed creation of C-S-H gel, which is a binding material very similar to the main product of the hydration of Portland cement, is primarily responsible for the recovery of strength and stiffness.

Anyway, some factors still need to be explored before alkaline silicate solutions can be confirmed as suitable healing agents for self-healing concrete applications. In particular, it has to be assessed if the expected reactions can actually be manifested in the specific conditions of encapsulated systems and how long would be needed for them to take place. The role of contact with air regarding the manifestation of the healing reactions needs to be clarified, since this interaction would be negligible in the case of encapsulated systems, where the soluble silicates are released directly into the cracks from the capsules dispersed in the material bulk. Furthermore, the age of concrete at the time of damage can play a role, since the amount of free calcium hydroxide available for the healing reactions tends to decrease in time with concrete aging.

The progression of the healing reactions in the conditions above described is quite difficult to characterize. Indeed, a destructive test would be necessary to analyze the chemical species and quantify the extent of the mechanical recovery, but on the other hand, it would compromise the integrity of the observed system, inhibiting the possibility to further monitor its evolution. Ultrasonic tests could be a possible alternative to estimate indirectly the occurrence of specific interactions between healing agents and air or healing agents and concrete matrix. Ultrasound might help to shed light on the duration of processes, such as gelification of the alkaline solution, and link such a duration to external factors, like the age of concrete, etc. This could for instance help to consider what factors are to be controlled or optimized in order to improve the effectiveness of the healing process.

Indeed, several parameters that can be measured in ultrasonic experiments are known to change when the microstructure of the investigated sample is evolving, as during healing, thus providing information on the evolving process itself. Two main classes of acoustic parameters could be identified, essentially as a result of linear and nonlinear acoustic experiments, respectively. On one side, linear acoustics has been extensively applied to investigate the presence and evolution of damage in concrete [21,22,23] and to monitor phase transition processes [24,25]. Among the various (linear) indicators, it is worth recalling the measurement of the transmission coefficient across a distributed crack region [26], the velocity variation [27], the softening-induced shift of the resonance frequency due to prestresses [28] or crack distributions [23]. On the other side, nonlinear ultrasonic methods have been recently proven to be even more sensitive than linear methods in order to monitor damage evolution (or even recovery due to autogenous healing mechanisms) in concrete [29,30,31,32,33]. Here, as well, several different indicators have been defined: second and third harmonics amplitude [22,34,35,36], the shift of the resonance frequency [37,38,39,40], coda wave properties [41,42] or the nonlinear indicator based on the Scaling Subtraction Method (SSM) [43,44,45,46]. They all have been widely used in the literature to monitor the evolution of damage and could be used to monitor structural transitions in the behavior of the healing agent in contact with a cementitious matrix.

In this paper, a combined linear-nonlinear ultrasonic approach was adopted to analyze the behavior of a cracked cementitious mortar in the presence of a thin layer of sodium silicate solution. The outline of the whole experimental setup and the characteristics of the specimens are described in Section 2, while the details of the single experiments are discussed in Section 3, together with the main results achieved. Verification of the mechanical recovery is discussed in Section 4, and concluding remarks are reported in Section 5.

## 2. Materials and Methods

### 2.1. Specimens

The specimens used for this study were in the shape of standard (4 × 4 × 16 cm${}^{3}$) mortar prisms. The mortar matrix was produced using a common CEM II/A-LL 42.5 R cement, a water-to-cement ratio of 0.5 and a cement-to-sand ratio of 1:3 by weight, in accordance with Standard UNI EN 196-1 Methods of testing cement, Part 1: Determination of strength. The age of the mortar prisms at the time of testing was about two years, thus guaranteeing that the cement hydration process was virtually completed and the amount of free calcium hydroxide stabilized.

The samples were preliminarily tested using the ultrasonic protocol described below in their “intact state”. Afterwards, a pass-through crack was generated at mid-span by means of three-point-bending tests up to failure, in such a way to create a complete disconnection between the two halves of each prism. A thin layer of sodium silicate solution was then applied manually to the crack surface, with the aim to reassemble the two residual fragments, thanks to the activation of the healing process, which would induce the progressive creation of a stable bond between the opposite edges of the fractured zone, bridging them together and eventually restoring the material integrity. The residual crack width after reassembling was evaluated by means of a 20× optical microscope, resulting in around 400 μm, on average. Such a residual crack width was deemed adequately representative of common damage states in real-sized concrete structures since, based on the indications reported in the European Standard EN 1992-1-1: Eurocode 2: Design of concrete structures—Part 1-1: General rules and rules for buildings, crack sizes of 200–400 μm (depending on the structure type and exposure class) have to be regarded as threshold values discriminating between acceptable damage in the serviceability limit state (cracks smaller than this do not need a specific control) and unacceptable crack widths (that require crack control).

In continuity with previous studies [8,47], the sodium silicate used was provided by Sigma Aldrich and was characterized by a 10.6 wt % proportion of Na${}_{2}$O, a 26.5 wt % proportion of SiO${}_{2}$ and a 62.9 wt % of water.

Intentionally, the geometric configuration of the specimens described above was rather simplified with respect to the real case of self-healing encapsulated cementitious systems. Indeed, the latter differs due to the presence of the ruptured capsules at the site of the crack and also because the healing agent released from the capsules does not necessarily flow up to covering the fracture surfaces entirely. However, in order to monitor the changes in the acoustic behavior of the interface between concrete and healing agent, this simplified system was easier to analyze because of the reduced number of testing variables which are not controllable. Furthermore, the chosen configuration was still realistic, in that it corresponded to the optimal diffusion of the silicate and the contact with air was limited to the borders of the silicate layer, as in real conditions.

### 2.2. Ultrasonic Testing Configurations

A set of three experiments was conducted one after another to analyze the acoustic and elastic properties of each sample. The cycle of three experiments was repeated at different times to monitor the effects of the evolution of self-healing during recovery. In particular, the system was monitored in the following states:

- in the initial state, referred to as “0” (i.e., when the prisms were still intact, before the creation of the transversal crack at mid-span via the initial three-point-bending test);
- in the damaged state, referred to as “1” (i.e., immediately after the generation of the mechanical disconnection at mid-span and before the application of the healing agent on the fracture surface);
- at regular time intervals denoted as “2” to “n”, during the healing process. The first set of measures during healing was taken a few minutes after the healing process has started, and the monitoring procedure continued up to three weeks; though major changes in the acoustic behavior turned out to be established in the first few days, as will be detailed in the following.

Mechanical testing was also performed after three weeks.

At each time, as mentioned, three experiments were performed to characterize both the linear and nonlinear elastic properties of the system. The three experiments, differing for the type of excitation source used, will be described in detail in Section 3. Here, it is sufficient to mention that the characterization consisted of:

- Resonant modes analysis under frequency sweep excitation;
- Linear and nonlinear analysis (transmission coefficients and harmonics generation) under continuous wave excitation;
- Nonlinear analysis according to the Scaling Subtraction Method (SSM) under pulse excitation.

### 2.3. Ultrasonic Testing Experimental Setup

**Figure 1:**
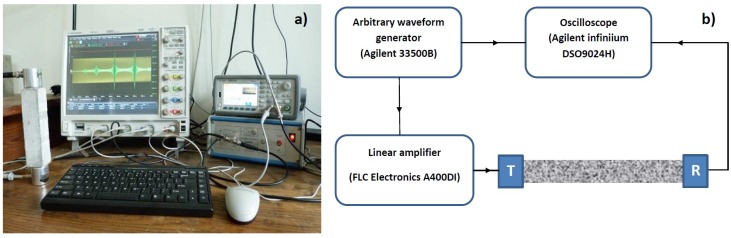
Experimental setup: (**a**) Photograph of the implemented configuration; (**b**) Sketch of the electronic configuration.

In all cases, the experimental configuration for the ultrasonic measurements was the same (see Figure 1): the specimens were equipped with two identical narrowband piezoelectric transducers (Matest C370-02), characterized by a central frequency of 55 kHz, a bandwidth of 5 kHz and a sufficiently flat (though necessarily attenuated) response in the frequency range out of the baseband bandwidth. They worked one as an emitting source and the other as a receiver. They were glued to the 4 by 4 cm${}^{2}$ opposite sides of the prisms by means of phenyl salicylate (a coupling agent with excellent linearity) and were not removed for the entire duration of the testing campaign.

The emitting transducer was connected to an arbitrary waveform generator (Agilent Technologies 33500B, Santa Clara, CA, USA), in series with a 20× fixed gain high voltage linear amplifier (FLC Electronics A400DI, Partille, Sweden), so that it was forced to vibrate according to a prescribed excitation function (different for the three characterization protocols). In parallel, it was connected also to a 4-channel digitizing oscilloscope (Agilent Technologies DSO9024H Infiniium), so that the input signals could be recorded and used to trigger the data acquisition. The receiving transducer was directly connected to the oscilloscope for data acquisition.

The linearity of the acquisition system was tested and verified in [33]. The same study reports the robustness of many of the ultrasonic methods used here with respect to small temperature and humidity fluctuations, as occurring in indoor conditions. Based on this conclusion, it was decided here to perform the ultrasonic testing without imposing any control of temperature and humidity, whose values ranged approximately between 19 and 24 ${}^{\circ }$C and 35% and 45%, respectively.

### 2.4. Mechanical Testing

All of the specimens were subjected to mechanical tests by means of three-point-bending immediately after the initial ultrasonic tests and at the end of the healing process, in such a way so as to determine the flexural strength in the original state and after repair. This testing procedure was used to confirm the information about the settling of the healing reactions and to formulate a final judgment on the quality of the repair process. The tests were performed using a 25-kN servo-controlled universal testing machine working in deflection control, with a test velocity of 0.015 mm/s.

## 3. Ultrasonic Tests: Results and Discussion

### 3.1. Resonant Modes Analysis under Frequency Sweep Excitation

A first set of ultrasonic experiments was performed in order to analyze the resonant modes of the specimens under a frequency sweep excitation. For the resonance experiments, the excitation function was set to a sine sweep ranging from 1–30 kHz in 1 s. A linear sweep was chosen. In this way, it was possible to excite the first three longitudinal vibration modes of the intact specimen (centered around 8, 16 and 24 kHz, respectively) and observe their evolution in time, from the intact state to a repaired configuration. The response of the specimens to such a sweep excitation was recorded using a time window of 1 s with a sampling rate of 400 kSa/s. Measurements were performed using a fixed amplitude of excitation $A_{sweep}=12$ V.

**Figure 2:**
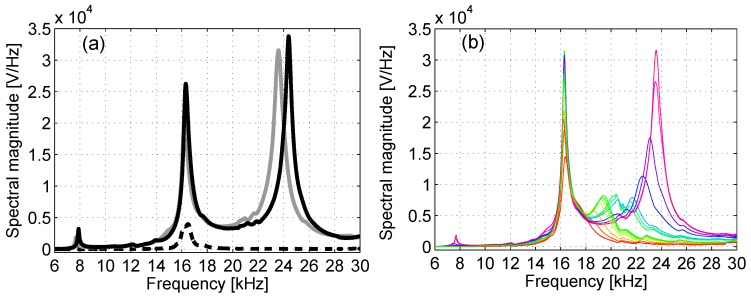
Evolution of the output signal spectra as a function of time. Data refer to Specimen No. 1. (**a**) Spectra at time “0” (intact state, solid black line), time “1” (damaged state, dashed black line) and time “n” (repaired state, solid gray line); (**b**) Spectra during the healing process, from time “2” to time “n”.

For each measurement, the spectral response was calculated based on the recorded data by means of a fast Fourier transform algorithm implemented through the MATLAB^®^ FFT function (with Blackman windowing and $4\times 10^{5}$ points, corresponding to the entire duration of the signal). Results are shown in Figure 2, where the evolution of the spectral magnitude with the progression of the healing process is shown for Specimen No. 1. A similar behavior was manifested by the other tested specimens also, but it was not reported here for the sake of brevity.

The spectra for the intact and damaged samples are shown in Figure 2a, together with the spectrum at the end of the healing process. It is easy to distinguish a sharp difference in the spectral response of the specimen between time “0” (intact state, solid black line) and time “1” (fully-damaged state, dashed black line). At time “1”, the sample presents a crack crossing the entire transversal section, and as a consequence, transmission is strongly affected with a much smaller vibration energy transmitted at time “1” with respect to time “0”. As a result, most of the modes disappear within the noise level, and only the peak corresponding to the mode at 16 kHz could be appreciated. Note that, since cracking occurs roughly in the middle of the sample, this mode is amplified with respect to the others, because it corresponds to the first resonance mode of the two halves of the sample: indeed, the crack reduces the free vibration length to half of the initial one, the first resonance frequency being redoubled in turn. After approximately five days (solid gray line), the spectrum shows again all peaks as in the intact state, with almost the same amplitude and slightly different resonance frequencies.

Data recorded at successive time intervals from “2” to “n” (Figure 2b) showed an intermediate behavior, with a progressive transition from a state similar to that of the cracked specimen up to a state very close to the intact one. It is worth pointing out that the measurement taken at time “2” (red line, corresponding to the lowest amplitude of the peak at 16 kHz) was performed a few minutes after the application of the sodium silicate solution to the fracture surface, and yet, evidence of a much improved signal transmission can be noticed (as will also be discussed later). Even though the healing agent is still in a completely fluid configuration, the magnitudes of the spectral peaks were significantly higher than at time “1” (though lower than at time “0”), and resonance peaks begin to appear close to 8, 16 and 24 kHz (corresponding to the peaks for a full-length specimen) as in the case of the intact specimen.

Both peaks at 8 kHz (slightly visible because of their low amplitude) and 24 kHz show the same behavior: immediately after application of the healing agent, some energy begins to be detected at the two frequencies, but without evidence of a clear peak. Later, when solidification of the healing agent is partially completed, the spectrum assumes a clearer shape (blue line) with peaks occurring at a frequency that increases (hardening) with the solidification process up to getting close to that of the intact state.

Slightly different is the behavior of the peak at 16 kHz (which corresponds to the second mode of the specimen and the first mode of each of the two halves). The frequency of the peak is rather insensitive to the evolution of the healing agent, while its amplitude seems to be diminishing with the solidification of the interfacial layer between the two halves of the sample, a consequence of the fact that the resonances within the two parts of the sample become less and less important.

**Figure 3:**
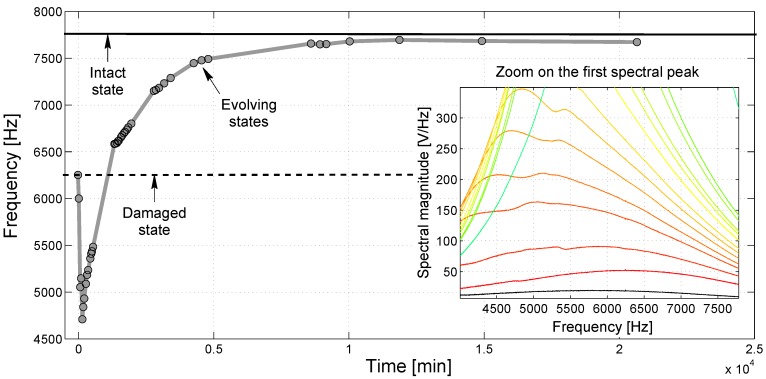
Resonance frequency of the first longitudinal mode as a function of time detected with a sweep source. Data refer to Specimen No. 1.

A more accurate observation of the spectral response during the process of sodium silicate hardening was performed for the peak at 8 kHz and Specimen No. 1 (see Figure 3). A similar behavior can be appreciated for the other specimens and for the peak at 24 kHz, as well, though not reported here for the sake of brevity. In Figure 3, the frequency at which the first mode is detected (i.e., the frequency of the maximum of the spectrum around 8 kHz) is shown vs. time. Apart from a drop towards lower frequencies occurring in the first few hours after the application of the sodium silicate solution (that could be ascribed to the presence of humidity supplied by the aqueous solution itself), the resonance peak moved towards higher values, eventually tending to the resonance frequency detected for the intact state. This resonance shift process seemed to show an asymptotic evolution, which can be considered substantially completed in about seven days (about 10${}^{4}$ min) after the beginning of the healing process.

In the inset, a zoom of the spectrum around 8 kHz is shown for the first instances of the evolution to highlight the decrease in the frequency at short times. Note that as long as the sodium silicate is still in a liquid configuration, the peak is far from being sharp.

Based on the discussion reported above, a first conclusion can be drawn: the acoustic response of a cracked cementitious mortar element, and in particular, its resonance behavior, changes in time due to the presence of a layer of sodium silicate in adherence to the fracture surfaces. Its evolution seems to converge towards the response originally manifested in the intact state, i.e., prior to crack formation, thus indicating a successful healing of the sample as long as its elastic/acoustic properties are concerned.

### 3.2. Linear and Nonlinear Analysis under Continuous Wave Excitation

**Figure 4:**
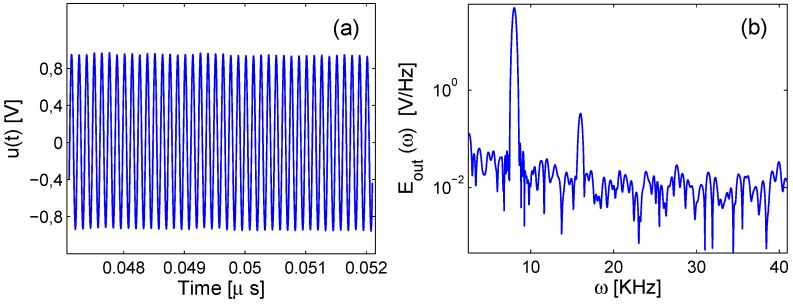
(**a**) Typical signal recorded with a sinusoidal source function and (**b**) relative spectrum.

A second testing procedure was implemented changing the type of excitation, which was set to a continuous sine wave with frequency of 8 kHz and an amplitude of 3 V. The sampling rate was set to 5 MSa/s. The response signal u(t) was detected once standing wave conditions were reached, and a fast Fourier transform of the signal was performed. A typical signal at a given healing time is shown in Figure 4a, and its spectrum is shown in Figure 4b. Note that the experiments under such a continuous wave excitation have to be regarded more precisely as a combination of transmission and resonance conditions, rather than pure transmission.

Two different indicators could be extracted from the spectrum. First, a sort of linear transmission indicator could be defined, related to the energy transmitted across the interface and detected at the receiver position. To estimate the transmission properties, the spectral magnitude of the output signal was integrated in a frequency window of δ=1 kHz centered around the frequency of the excitation wave ($\omega_{0}=8$ kHz):

(1) $$E_{\omega }=\int_{\omega_{0}-\delta /2}^{\omega_{0}+\delta /2}E_{out}\left(\omega \right)d\omega$$

The transmission coefficient *T* is defined by dividing $E_{\omega }$ by the spectral amplitude of the input signal integrated in the same time window:

(2) $$T=\frac{E_{\omega }}{E_{\omega }^{inp}}=\frac{\int_{\omega_{0}-\delta /2}^{\omega_{0}+\delta /2}E_{out}\left(\omega \right)d\omega }{\int_{\omega_{0}-\delta /2}^{\omega_{0}+\delta /2}E_{inp}\left(\omega \right)d\omega }$$

It has to be pointed out that the frequency of the excitation and hence the central frequency value at which the output contribution was evaluated were intentionally set to 8 kHz in order to enhance the perceptibility of the output signals by improving the signal-to-noise ratio, since this frequency corresponded roughly to the first resonance mode of the intact specimens, as discussed in Section 3.1.

**Figure 5:**
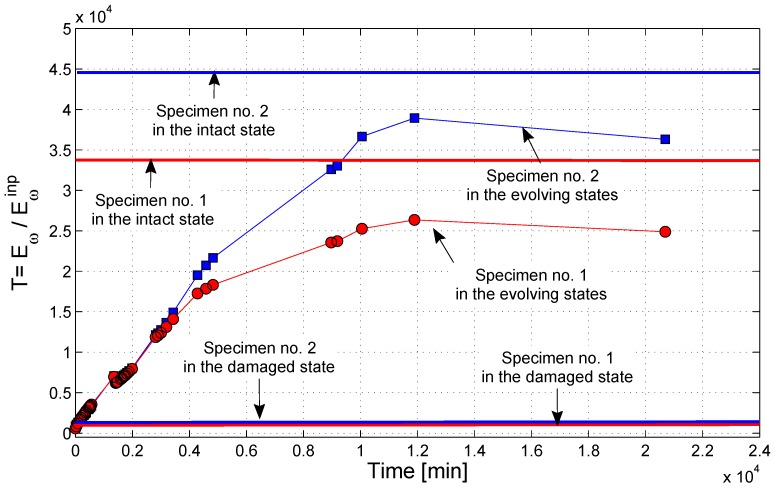
Evolution in time of the transmission coefficient *T* at the fundamental frequency for Specimen No. 1 and Specimen No. 2.

The evolution in time of T is illustrated in Figure 5 for two of the analyzed samples. The values of the transmission coefficient for the intact and damaged samples (states “0” and “1”) are reported as solid lines. It is easy to observe an asymptotic behavior during healing: indeed, T increased in time from a very low value immediately after the application of the sodium silicate solution to the fracture surface up to a steady value that was reached again in about seven days (10${}^{4}$ min). The final value of the transmission coefficient is very close to that of the intact sample. The same evolution with the same time scale was captured through the resonance experiments (see Section 3.1). Therefore, apparently the sodium silicate was able to interact with the cracked cement matrix from a physical-chemical point of view, and as a consequence, the acoustic behavior of the cracked specimens changed in time as long as the healing reactions due to the presence of the alkaline solution were taking place, with a progressive recovery of the acoustic properties initially shown by the intact specimen. The increase of the output spectral magnitude in the transmission experiments, as well as the spectral changes in the resonance experiments were proofs of such a healing phenomenon.

The spectrum of the signal shown in Figure 4b shows also the emergence of a nonlinear effect, that is the generation of higher order harmonics. In particular, attention was focused here on the analysis of the second order harmonic, i.e., the peak at a frequency value doubled with respect to the fundamental one (16 kHz). As usually observed, the higher the excitation amplitude, the more evident was the nonlinear harmonic generation phenomenon (as will be discussed later). To quantify the effect, it is possible to define a parameter $E_{2\omega }$ representing the amplitude of the second harmonic, obtained integrating the spectrum around the second harmonic peak in a similar way to Equation (1). This quantity, divided by the fundamental harmonic amplitude to avoid spurious effects from the increase of the transmission coefficient discussed above, allows one to define a nonlinear parameter:

(3) $$E_{NL}=\frac{E_{2\omega }}{E_{\omega }}=\frac{\int_{2\omega_{0}-\delta /2}^{2\omega_{0}+\delta /2}E_{out}\left(\omega \right)d\omega }{\int_{\omega_{0}-\delta /2}^{\omega_{0}+\delta /2}E_{out}\left(\omega \right)d\omega }$$

**Figure 6:**
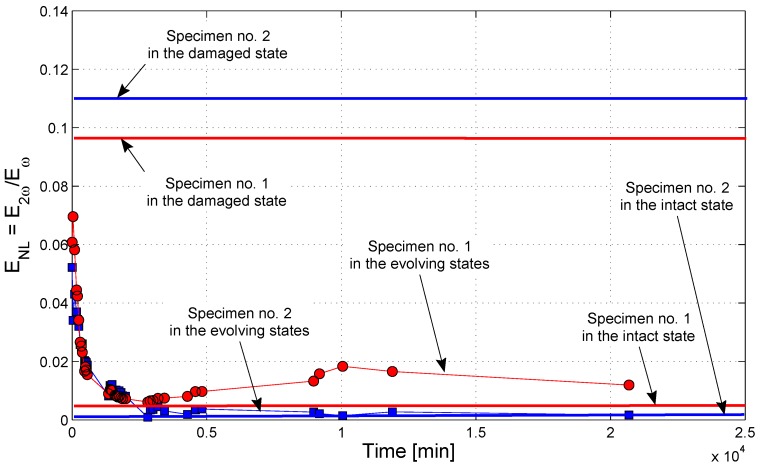
Nonlinear effect of second harmonic generation under continuous sine excitation as a function of healing time for Specimen No. 1 and Specimen No. 2.

The parameter $E_{NL}$ is shown vs. time in Figure 6 for two samples. The values for the intact and damaged states are reported as solid lines for reference. It is remarkable that the nonlinear effect tended to disappear in time with the progression of sodium silicate hardening: the amount of harmonics generated at time “0” corresponds roughly to the maximum of harmonics generation, as observed on the cracked sample, while it reduces to substantially the same value as the intact sample at the end of the process. The observation is in counter trend with respect to the spectral magnitude corresponding to the fundamental frequency of 8 kHz. In principle, one would expect second harmonic generation to be enhanced by the improved transmission, since in this way, a higher exciting signal is able to reach the fractured region. The result obtained goes in the opposite direction. Therefore, since the appearance of nonlinear effects is known to be related to the presence and the extent of damage, the reduction of nonlinear effects has to be interpreted as a sign of the partial recovery of the material integrity at the fractured interface. Both the solidification of the healing agents (solids in general exhibit less nonlinear features than fluids) and the reduction of clapping between the concrete surfaces are indeed contributing to the reduction in harmonics generation.

A non-negligible difference in time scales with respect to the results of the linear measurement discussed above is however noticeable. The stabilization of the specimens response was reached in approximately four days (about 5000 min), as visible in Figure 6. This could indicate that the healing process is actually a multifaceted phenomenon that involves changes in viscosity of the silicate solution in parallel to chemical reactions with the cement matrix, with the possible formation of different chemical species at different times (see also Section 3.4). The nonlinear analysis is able to capture the variations in the material microstructure that occur in the earlier stages of such a complex healing process, while the linear analysis is probably more suitable to describe the macroscopic changes that finally lead to a mechanical recovery.

**Figure 7:**
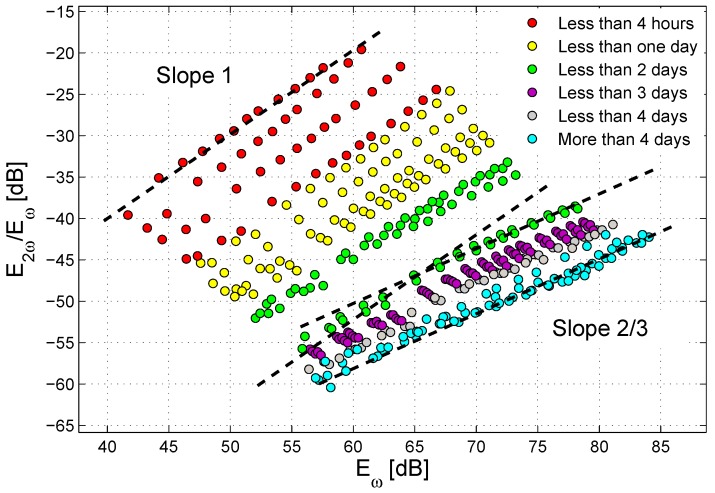
$E_{NL}=E_{2\omega }/E_{\omega }$ versus $E_{\omega }$ for variable-amplitude sine excitations as a function of healing time for Specimen No. 1.

The nonlinear analysis discussed above was further developed with an additional test, in which the amplitude of the exciting sinusoidal wave was progressively increased in the range 50 mV–10 V, and the dependence of the harmonics generation as a function of the strain in the sample was analyzed. This corresponds to a standard Nonlinear Elastic Wave Spectroscopy (NEWS) approach [48]. At each time during the healing process, the quantity $E_{2\omega }/E_{\omega }$ is plotted versus $E_{\omega }$ (see Figure 7). The results found are in agreement with previous studies, which have reported that the output amplitude $E_{NL}$ corresponding to the second harmonic component normalized to the output amplitude at the fundamental frequency has a power-law dependence on the output amplitude $E_{\omega }$ at the fundamental frequency [48,49]:

(4) $$E_{NL}=\frac{E_{2\omega }}{E_{\omega }}=aE_{\omega }^{b}$$

The power-law behavior can be easily appreciated using a dB scale as in Figure 7, where the power-law correlations between the two quantities are represented by straight lines, the slope of each corresponding to the respective exponents in the power-law plot. The generation of the second harmonic (at each given time) increases with increasing strain energy in the sample (each value of $E_{\omega }$ corresponds to a given excitation amplitude). Once again, it is possible to notice that the extent of the nonlinear phenomena tended to decrease in time (curves flattening to the right-bottom side of the plot). In addition, the slope of the curves showed a variation in time, changing from a slope close to one for the initial measurements to a slope approximately equal to 2/3 at the end of the healing process, in analogy with some cases reported in the literature [50].

Quite surprisingly, the curves corresponding to the measurements performed in a time interval between one day and two days after the application of the sodium silicate to the fracture surface displayed a double slope, meaning that two different slope values seemed to be suitable to fit the data for low excitation amplitudes and high excitation amplitudes, respectively. This phenomenon has already been observed in the literature for non-consolidated granular media [51,52].

### 3.3. Nonlinear Analysis According to the Scaling Subtraction Method under Pulse Excitation

**Figure 8:**
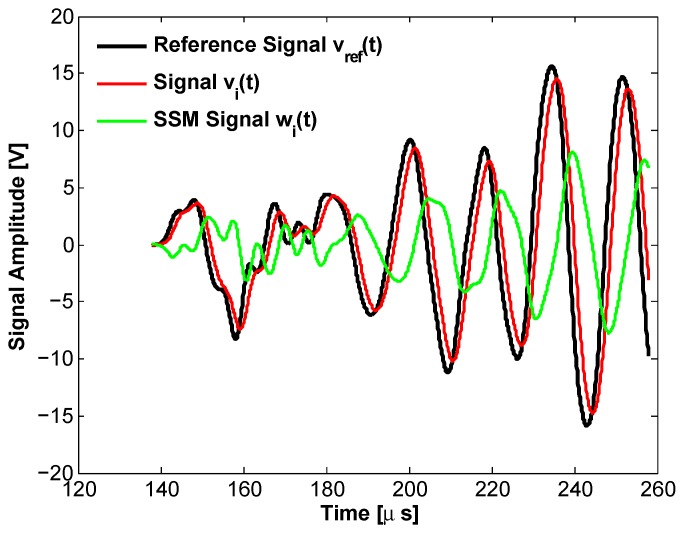
Example of reference signal $v_{r}ef\left(t\right)$ (black line), detected signal $v_{i}\left(t\right)$ (red line) and scaled-subtracted signal $w_{i}\left(t\right)$ (green line) in a short time window close to the first arrivals time. Data refer to a sample at an intermediate level of healing and intermediate excitation amplitude $A_{i}=12$ V.

A final nonlinear analysis using pulse excitations at various amplitudes was implemented to complete the ultrasonic investigation on the effect of a sodium silicate solution on a cracked cementitious matrix. It was conducted in accordance with the Scaling Subtraction Method (SSM) procedure and therefore allowed taking into account the contributions to nonlinearity due not only to higher order harmonics generation (see Section 3.2), but also to the nonlinear attenuation and phase shift phenomena, as pointed out in [43]. As fully detailed in previous works [30,44,45,53], the SSM protocol consisted of:

- exciting the specimens using a sequence of pulses at various amplitudes $A_{i}$ in the range 50 mV–15 V (by means of the same equipment described in Section 2.3). More specifically, a rectangular pulse of width t=10μs was used;
- at each time from “2” to “n”, recording the output response $v_{i}\left(t\right)$ of the specimen to such a variable-amplitude excitation (in a time window of 10 ms, with a sampling rate of 10 MSa/s);
- calculating the so-called “reference signals” at injection amplitude $A_{i}$, defined as $v_{ref}\left(t\right)=A_{i}/A_{1}v_{1}\left(t\right)$ where $v_{1}\left(t\right)$ is the signal detected at the lowest excitation amplitude $A_{1}$;
- computing the “scaled-subtracted signals” obtained as the difference in time between the actual output signals at the various excitation amplitudes and the reference signals at the same amplitudes: $w_{i}\left(t\right)=v_{i}\left(t\right)-v_{ref}\left(t\right)$; examples are reported in Figure 8;
- summarizing the information contained in the whole temporal signals using compact indicators: in this case, in continuity with [33], the root mean square (RMS) of the signals (v(t) and w(t)) over a prescribed time interval including only the first arrivals was used. It represents the average power of the signals over the assigned time interval and was denoted as *x* (RMS of the output signal v(t)) or *η* (RMS of the scaled-subtracted signal w(t)), while the ratio η/x was referred to as *y*;
- analyzing the relation between *y* and *x* and its evolution in time, thus providing information on the type and extent of nonlinearity in the system as a function of the progression of the healing process.

The signals $v_{ref}\left(t\right)$ (black line), $v_{i}\left(t\right)$ (red line) and $w_{i}\left(t\right)$ (green line) are shown in Figure 8 for $A_{i}=12$ V. It is possible to observe the nonlinear phase shift and attenuation that make the signal $v_{i}\left(t\right)$ different from $v_{ref}\left(t\right)$. The SSM approach allows one to define a nonlinear signal $w_{i}\left(t\right)$, which presents a good signal to noise ratio (much higher than harmonics).

**Figure 9:**
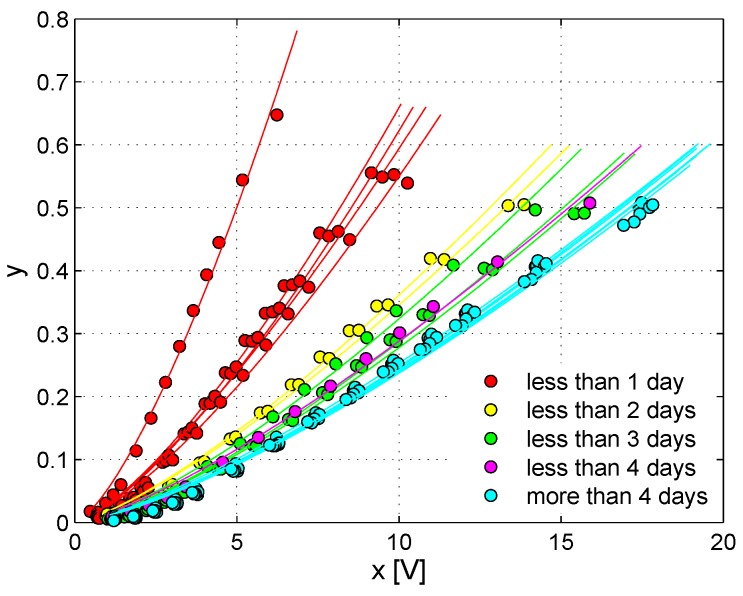
*y* versus *x* plot for variable-amplitude pulse excitations in Scaling Subtraction Method (SSM) experiments at different healing times.

The results of the SSM analysis confirmed the conclusions achieved through the previous nonlinear experiments (see Section 3.2). Plotting *y* vs. *x* (Figure 9) revealed that nonlinear phenomena, globally including secondary harmonic generation, nonlinear attenuation and phase shift, were present in the system because the nonlinear indicator *y* extracted from the scaled-subtracted signals increases with amplitude; thus, the difference between reference and recorded signals is not due to noise effects alone [54].

Nonetheless, such a nonlinear behavior decreases significantly in time, as proven by the shifting of the *y* versus *x* curves to the right-bottom side of the plot, meaning that the material was actually experiencing a consolidation in time due to the occurrence of the healing process, so that the effect of the crack, which was primarily responsible for the nonlinear manifestations observed during the experiments, had to be considered as negligible at the end of the healing process. A stabilization in the nonlinearity decrement seemed to be reached in approximately five days (about 7000 min), in good accordance with the results reported in Section 3.2.

**Figure 10:**
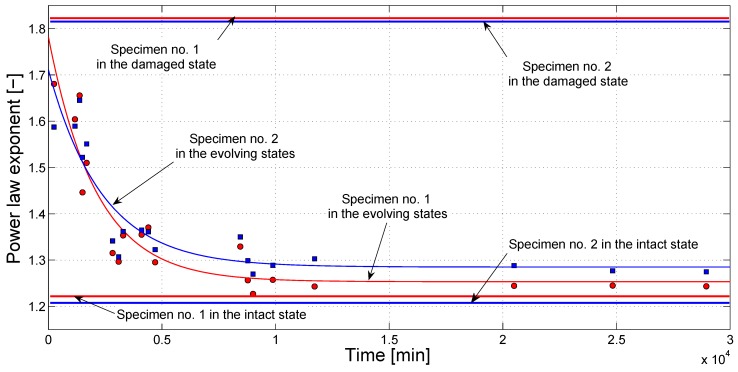
Time evolution of the exponent of the power-law fitting used to interpolate the *y* versus *x* data from SSM experiments.

This behavior is similarly visible when considering the evolution in time of the exponent of the power-law fitting that can be used to interpolate the *y* versus *x* data [48,50,51,55]. As highlighted in Figure 10, the exponent decreases in time of about 15%–20% and reaches a plateau in approximately four days (same as inferred from nonlinear harmonics measurements). This behavior is qualitatively similar for all specimens analyzed and is reported in Figure 10. The exponent found at the end of the healing process is about 1.2, very close to the exponent found from measurements in the intact case, reported as a reference as a solid line. The exponent at the beginning of the healing process is only slightly smaller than the exponent measured on the cracked sample (again reported for reference as a solid line).

### 3.4. Discussion

On the whole, the experiments performed point out two relevant results, concerning (a) the time scale over which significant events occur and (b) the indicators of the amount of damage in the sample. In all cases, the recovery of the system seems to be clearly assessed, at least as concerns the recovery of the elastic and acoustic properties. In all figures reported above, both linear and nonlinear indicators show that:

- Immediately after applying the sodium silicate solution, the value of all of the indicators is very close to that of the broken sample;
- After a few days, the healing agent is supposedly almost completely solidified, and the ultrasonic parameters are very close to those measured on the sample in its initial intact state.

As concerns the time scales, acoustic recovery seems to be completed within a few days, i.e., in a much shorter time than expected for full mechanical recovery (20–30 days, according to literature data). The time scale for the recovery of the nonlinear properties seems slightly shorter than that for the recovery of the linear elastic properties: 4–5 days vs. seven days. At this stage, the meaningfulness of this difference has still to be confirmed and analyzed. Anyway, a possible interpretation could be found in the essentially different mesoscopic elements mostly influencing nonlinear vs. linear processes in similar systems. It has been shown for instance in [52] that the weakest contacts in a granular assembly (thus, the smallest and softest elastic elements in the system) are mostly responsible for the nonlinear elastic properties, while the strongest contacts mostly define the structure strength and linear properties. More recently, in [56], the relaxation effect of slow dynamics has been analyzed, which can be regarded, to some extent, as a self-healing process of elastic property recovery: here, the essentially different roles of weak contacts and strong contacts are highlighted in the different time scales involved in the multiple relaxation processes. Therefore, in the present healing process, it can be speculated that the two time scales for nonlinear and linear elastic parameters’ recovery correspond to the onset and evolution of different chemical-physical processes, e.g., gelification and penetration into the cracks (soft contacts) and the formation of solid reaction products (hard contacts).

The evolution of the exponent of the power-law expressing the dependence of the nonlinear indicator on the excitation amplitude (Figure 7 and Figure 10) also could give information about the physical processes occurring in the sample. In all of the cases here examined, the power-law exponent decreases with healing time. Elsewhere, it has been shown that an increase of the power-law exponent was possibly ascribed to the appearance of open cracks in a sample [50]. Thus, the results shown here seem to indicate that the sodium silicate here used as a healing agent contributes significantly to crack closure. This effect, taking place in a quite short time, could be an indication of the good quality of the healing agent chosen.

## 4. Mechanical Tests: Results and Discussion

As anticipated in Section 2.4, at the end of the ultrasonic testing, the specimens were subjected to a final three-point-bending test to determine their flexural strength after repair in comparison to the initial one. Such a mechanical test being destructive, it could not be performed at different times during the healing process as the ultrasonic measurements, but it had to be executed just once, when the acoustic response was proven to be unquestionably stabilized. As a matter of fact, it was performed three weeks after the generation of the crack at mid-span and the subsequent beginning of the healing process. Such a decision was made because from the literature data, it was inferred that at least three to four weeks is the curing time normally required when using sodium silicate in concrete applications, for example as a sealant for concrete pavements. For the same reason, the ultrasonic observation was globally prolonged for three weeks, as well, though stabilization of the acoustical properties turned out to be ensured in a much shorter time.

**Figure 11:**
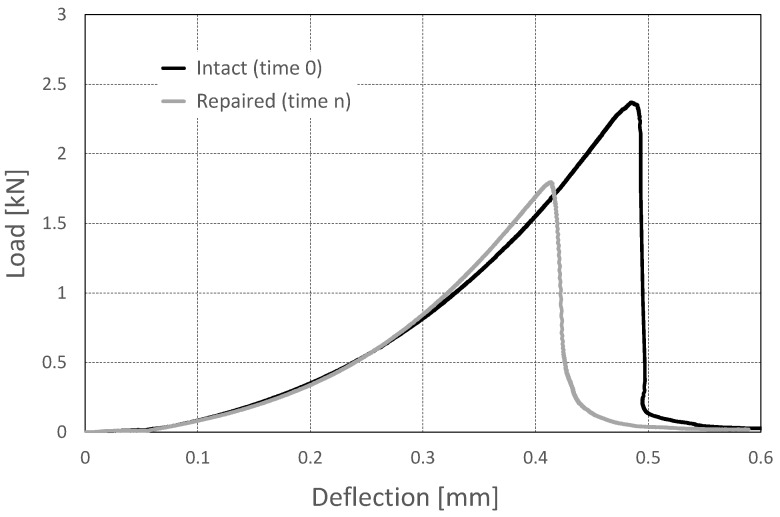
Load versus deflection curves from three-point-bending tests in the intact state (solid black line) and in the damaged state (solid gray line).

An example of the mechanical results achieved is reported in Figure 11. Here, the load versus deflection curve for one of the repaired prisms at the end of the healing process is shown in comparison with the one initially obtained for the same prism in the intact state, as measured during the loading process applied to break the prism into two halves. All of the repaired specimens showed a very satisfactory mechanical performance, with a load recovery in the range 66%–76% of the original strength.

**Figure 12:**
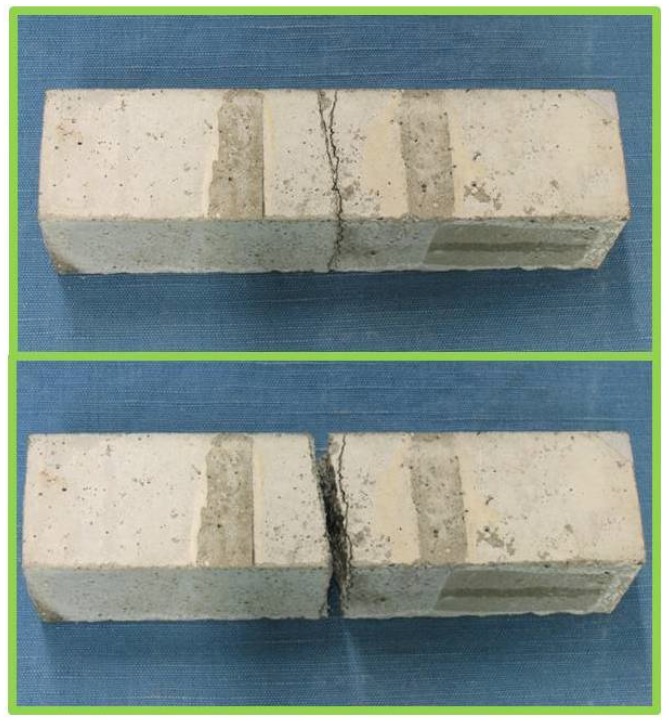
Detail of one of the specimens after the final three-point-bending test, with the creation of a new crack path partially separated from the one generated via the first flexural test.

The recovery was even more impressive when observing the aspect of the repaired specimens at the end of the flexural tests: as visible in Figure 12, a new crack path was created during the second three-point-bending test, which only partially coincided with the one previously generated at the beginning of the testing campaign. The recovery was limited to a fraction of the original strength probably because the manual application of the sodium silicate to the fracture surface was imperfect at the edges, thus possibly reducing the active cross-section in the repaired sample with respect to the intact one. However, on the whole, the mechanical tests provided clear evidence of the effectiveness of sodium silicate as a healing agent, since the fractured/repaired region perfectly resisted a subsequent flexural test up to failure, both from a qualitative and a quantitative point of view.

## 5. Conclusions

The interaction between a cracked cementitious matrix and a solution of sodium silicate was analyzed in terms of acoustic behavior by means of linear and nonlinear ultrasonic techniques. The alkaline solution was used as a repairing agent to consolidate a pass-through crack deliberately generated at mid-span of some mortar prisms by means of three-point-bending tests.

The linear ultrasonic analysis investigated the resonance modes and the acoustic transmission properties of the prisms in their intact, cracked and progressively repaired states, revealing that some changes were induced in time by the presence of the sodium silicate and that the healing mechanism thus created was able to virtually turn the acoustic behavior of the prisms back to their initial state, prior to crack formation. The nonlinear analysis by means of NEWS and SSM confirmed such effects, also allowing one to speculate about the kinetics of the healing reactions induced by the alkaline solution, though the complexity of the healing mechanism itself and the impossibility to perform destructive characterizations during the healing process make it difficult to provide an exhaustive explanation, and further investigation is still to be done.

The flexural tests performed at the end of the healing process revealed a satisfactory recovery of mechanical properties, up to 76% of the initial strength in just three weeks. This finding not only confirms the suitability of sodium silicate to be used as a repairing agent for self-healing concrete applications, but also validates the ability of the ultrasonic techniques here used to detect the significant microstructural changes that eventually affect the mechanical behavior.

The relation between the recovery time for the acoustic properties (here observed to be restored in 5–7 days) and the recovery time for the structural properties of the sample (here tested at 21 days) still needs to be further explored. Additional investigations of the ultrasonic behavior in a long time range (more than three weeks), together with an early mechanical testing of the structural recovery (at 5–7 days) are planned in order to shed light on this issue. Linking the recovery behavior of the acoustic properties to the final mechanical recovery will eventually allow one to:

- define a measure of the expected final recovery without the need for a destructive testing;
- speed-up the optimization of the healing agents by sensibly reducing the time needed to assess the recovery in different conditions and for different healing agents;
- define a protocol to ultrasonically monitor the healing process when the healing agent is released from broken capsules, as in real conditions.
